# Visualization of oligomerization, clustering, and density transition of intrinsically disordered proteins

**DOI:** 10.1093/nsr/nwag107

**Published:** 2026-02-14

**Authors:** Jia-Ye Li, Yi-Hao Niu, Yi-Qin Gao, Huan Wang

**Affiliations:** Beijing National Laboratory for Molecular Sciences, College of Chemistry and Molecular Engineering, Key Laboratory of Polymer Chemistry & Physics of Ministry of Education, Peking University, China; Beijing National Laboratory for Molecular Sciences, College of Chemistry and Molecular Engineering, Key Laboratory of Polymer Chemistry & Physics of Ministry of Education, Peking University, China; Beijing National Laboratory for Molecular Sciences, College of Chemistry and Molecular Engineering, Key Laboratory of Polymer Chemistry & Physics of Ministry of Education, Peking University, China; Beijing National Laboratory for Molecular Sciences, College of Chemistry and Molecular Engineering, Key Laboratory of Polymer Chemistry & Physics of Ministry of Education, Peking University, China; Beijing Advanced Center of RNA Biology (BEACON), School of Life Sciences, Peking University, China; National Biomedical Imaging Center, College of Future Technology, Peking University, China

## Abstract

This study directly visualizes how Intrinsically Disordered Proteins form condensates, revealing a multi-step nucleation process that begins with single molecules and proceeds through intermediate clusters and density transition.

Molecular interactions of proteins underpin all cellular processes. Intrinsically disordered regions/proteins (IDRs/IDPs) are abundant in membraneless organelles (MLOs) [1], yet their functions through dynamic and subtle interactions are not fully understood. Protein aggregation and phase transition are commonly described by classical nucleation theory (CNT), which effectively models processes in materials science. According to CNT, biomolecule clusters of radius *R* form due to thermal fluctuations with free energy $\Delta G( R ) = 4\pi {R}^2\gamma + \frac{4}{3}\pi {R}^3\epsilon $, depending on surface tension *γ* and free energy per unit volume $\epsilon $. The surface tension term is always unfavorable; the volume term becomes favorable when phase separation is thermodynamically favored, which occurs when the concentration exceeds the saturation concentration, *c*_sat_. Thus, a critical cluster size (nucleation barrier) exists, below which clusters shrink, and those above it grow [2]; however, it fails to explain how low-abundance proteins can become enriched within cells. Experimental evidence further indicates that cluster formation can involve multiple nucleation steps, suggesting the applicability of a non-classical nucleation theory (NNT). To explain the existence of clusters at solution concentrations below *c*_sat_, the concept of phase transition coupled to percolation (PSCP) has been proposed [3]. Current limitations in spatiotemporal resolution constrain advanced imaging techniques to either single-molecule observations or the dynamics of larger assemblies [4]. As a result, direct visualization that reconciles the discrepancies between CNT and NNT during the early stages of liquid-liquid phase separation (LLPS) is still lacking [5,6]. The diversity of theoretical and experimental findings makes it a compelling research challenge to understand how protein phase separation nucleates—from individual molecules to small oligomers—and evolves from molecular clusters to dense phases with radii exceeding several tens of nanometers.

Utilizing liquid-phase transmission electron microscopy (LP-TEM) with a graphene liquid cell (GLC) geometry [7] (see experimental procedures in Text S2 and technical details addressing effects of electron beam, graphene surface and protein concentration in Text S3 and Fig. S1), we tracked the progression from IDP molecules (several nanometers in size) to clusters (10–20 nm) and finally to a dense phase (several tens of nanometers in radius). This behavior was consistently observed across the low-complexity domain of fused in sarcoma (FUS-LCD), full-length FUS (FL-FUS) and Tau protein. Clusters were found to form both below and above *c*_sat_, with density transitions ultimately leading to compact dense phases. During oligomerization, IDP conformation remained largely unperturbed as clusters assembled. In contrast, as these clusters transitioned into a dense phase, the IDPs underwent conformational changes to reduce repulsion. By resolving nanoscale morphological differences, our data uncover a multi-step nucleation mechanism that highlights deviations from classical homogeneous nucleation pathways.

Beginning with individual mole-cules, stepwise oligomerization from single molecules triggered a density transition upon reaching a critical number. Protein concentration governed the transition which is reproducible across salt and viscosity changes (Text S4, Fig. S2 and Movies S1–S3). We observed that clusters exist both above and below *c*_sat_ (1 µM) [8]. The formation of clusters from individual molecules is presented in Fig. 1a and Movie S4. First, the monomer size was determined based on the size of a single FUS-LCD molecule, consistent with molecular dynamics (MD) results (see details in Text S5). As a general mechanism, individual proteins initially associate to form small oligomers—such as dimers and trimers, identified in LP-TEM images by two or three distinguishable subunits and projected areas approximately twice or three times that of monomers (Text S6, S7 and Fig. S3), respectively—which subsequently assemble into larger clusters through further recruitment. This process is illustrated here using FUS-LCD as a representative example. Trajectory analysis (at 12.00 s in Fig. 1a) revealed long-range, directional movements driven by multivalent cation–${\mathrm{\pi }}$ interactions [9], accompanied by molecular reorientation.

**Figure 1.**
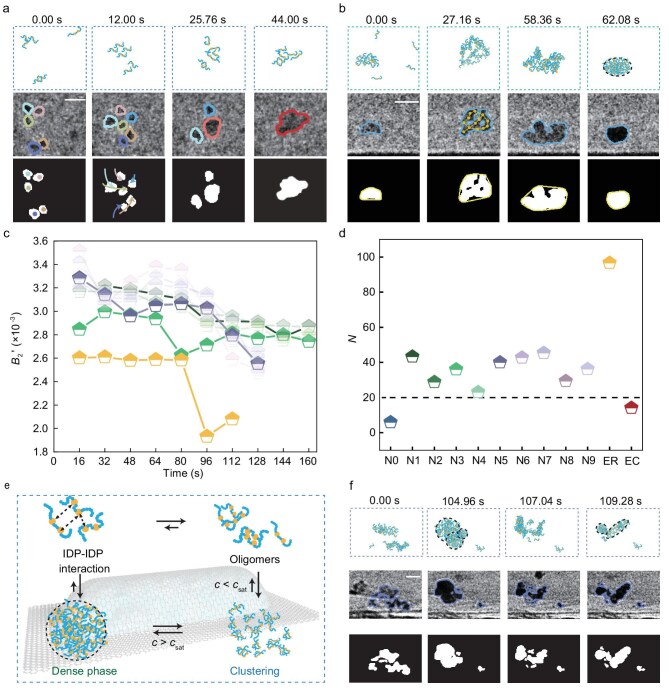
Clustering of FUS-LCD in solutions imaged by LP-TEM. (a and b) Time-resolved images showing the interaction between oligomers (a) and representative density transitions (b). First row: schematic illustrations (blue stickers, spaced by yellow spacers, form oligomers that assemble into networks, ultimately transitioning into a dense phase that we drew based on the LP-TEM images and the sticker-and-spacer model proposed by Pappu *et al*. [3]). Second row: LP-TEM images with circles highlighting the regions of interest. At 27.16 s in (b), yellow dots and lines indicate the network-like structures. Third row: binarized images analyzed using the UNet++ neural network. Trajectories of trimers (cyan, green, pink), dimers (purple, orange) and monomers (blue) were overlaid on the binarized images in (a). Yellow circles mark the periphery of the cluster in (b). Scale bars: 10 nm (a), 20 nm (b). (c) Temporal analysis of density transition videos (100 frames per segment) demonstrating a decrease in the second virial coefficient *B*_2_’ during the transition process. Green pentagons (dark to light) represent 4 density transitions N1–N4 in Movie S5, purple pentagons (dark to light) represent 5 density transitions N5–N9 in Movie S6, yellow pentagon represents the density transition, dispersion and reassembly process shown in (f) and Movie S7. (d) Critical molecule number *N* calculated at density transition point. The blue pentagon represents 6 molecules in (a) when only oligomerization occurs and the red pentagon represents one failed scenario in dilute solution with ∼600 nM FUS-LCD. Green, purple and yellow pentagons correspond to the samples in (c). (e) Schematic of FUS-LCD interactions forming from monomers into oligomers that further assemble into clusters, ultimately transitioning into a dense phase. (f) Time-resolved images showing the reversible interconversion of all forms in (e). Top to bottom: schematic illustrations, LP-TEM images with highlighted regions, and UNet++-analysed binarized images. Scale bar: 20 nm.

When the bulk concentration (∼6–60 µM) exceeded *c*_sat_ (1 µM), an interconnected network began to emerge (Fig. 1b). Clusters continued as oligomers incorporated into them, and their number increased over time. A density transition ultimately occurred (58.36–62.08 s) once the local concentration reached a critical threshold. ‘Projected area’ is the white-mask area in the binarized image (white-pixel counts × pixel area), and ‘contour area’ is the area inside the convex hull of all white-mask regions. Throughout cluster assembly, the projected area and intensity of individual IDPs remained constant (Fig. S4a). In contrast, during the density transitions, the initially interconnected structure (27.16 s in Fig. 1b) transformed into a nearly spherical dense phase (after 58.36 s in Fig. 1b), characterized by a reduced projected area (Fig. S4b), along with increased intensity and roundness (Fig. S4c). We define the sudden increase/decrease point as the density transition point: before it, the structure is a cluster; after it, it becomes a dense phase. The increased ratio of projected area to contour area indicated molecular rearrangement toward tighter packing (after 58.36 s in Fig. 1b). Although the exact internal concentration within GLCs fluctuated around the bulk concentration due to the stochastic nature of their formation, these processes were highly reproducible across different GLCs (Movies S5–S7) with quantitative analyses provided alongside the movie data, Fig. S4 and Text S7. In contrast, when the bulk concentration is below the saturation concentration (∼600 nM < *c*_sat_), interconnected clusters formed in the absence of density transitions.

To elucidate the factors driving the density transition and the emergence of a highly compacted state, we determined the second virial coefficient, *B*_2_′, using a Zimm plot analysis based on the scattering measurements, see details in Text S8. Of the measured states, oligomers exhibited the highest *B*_2_′ (∼0.18), which is about two orders of magnitude greater than values for clusters and dense phases ((1.9–3.5) × 10^−^^3^). This relatively large positive value indicates that these early species are highly solvated and remain in an expanded state due to conformational disorder and charge repulsion. Tracking the dynamic evolution during density transition revealed a statistically significant decline in *B*_2_′ from 2.9 × 10^–3^ to 2.6 × 10^–3^ (64–80 s in Fig. 1c). This reduction reflects decreased intermolecular repulsion, implicating conformational changes as a key driver of the process. Although Förster resonance energy transfer (FRET) experiments have previously associated conformational changes with phase separation [10], our data provide further mechanistic insight by delineating the specific pathway involved.

For the highly compacted dense phase, we estimated protein concentration based on the cross-sectional area of the monomer. Using this area, we calculated that the number of protein molecules per dense phase ranges from 20 to 100 (Fig. 1d), corresponding to an equivalent concentration from 19 to 110 mM, see details in Text S9. The threshold concentration of 19 mM aligns closely with the 27.8 mM measured via nuclear magnetic resonance (NMR) [9]. When the bulk concentration was below saturation, interconnected clusters formed in the absence of density transitions. Under these conditions, the maximum cluster size, *N*, was 14 (red in Fig. 1d), below the minimum *N* of 20 required for a density transition. Consistent with the absence of transition, *B_2_*′ remained initially constant before increasing slightly, indicating the fragmentation of clusters into smaller oligomers. This observation helps explain previous reports of cluster formation without phase separation at subsaturated concentrations [6].

Although highly concentrated and compacted, the dense phase remains dynamic. In addition to two dense phases fusing into a larger dense phase (Movie S8 and Fig. S5a), the intrinsic flexibility of IDPs facilitates transient, reversible interactions, enabling the coexistence of all states (Fig. 1e, Fig. S5b and Movie S9 in Text S10). Clusters can redisperse into oligomers at any concentration, and these oligomers may in turn reassemble into new clusters, thereby sustaining a cyclic process. Interconversion was also observed among dense phases: an existing dense phase could disassemble and later reassemble into a new one (104.96 s to 109.28 s in Fig. 1f and Fig. S4d), which could disassemble and subsequently reassemble into new dense phases (109.28 s in Fig. 1f), as indicated by concurrent decreases in both roundness and the projected area-to-contour area ratio. Notably, analogous dynamic equilibrium behavior was observed for both FL-FUS and Tau proteins (Movie S10). Although oligomerization and density transitions occur sequentially, we captured these processes in separate imaging sessions. This approach was necessary to minimize electron beam–induced protein damage, which would otherwise result from prolonged exposure.

We directly visualized density transitions of IDPs at the single-molecule level: an initially unfavorable monomer assembly nucleates clusters; subsequent conformational rearrangement drives a density transition that recruits additional monomers, revealing key intermediates of early LLPS nucleation. These observations substantiate a multi-step nucleation mechanism consistent with a first-order LLPS transition (Text S11) and account for the enrichment of low-abundance proteins, clustering under subsaturated conditions, and IDP-specific aggregation kinetics. Because pathological aggregation of neurodegeneration-related IDPs stems from rare, noncanonical events, our experiment resolves such heterogeneous dynamics while directly assessing targeted drugs and their mechanistic steps in intervening in LLPS. Moreover, density transitions occur only above a threshold concentration, governed by spatial organization and mass transport, which underpin LLPS functions in crowded cellular environments. The influence of the local electrochemical environment—including ion distributions and interfacial double layers—on condensate catalysis and interfacial properties can be elucidated through correlated LP-TEM, FRET measurements, and electrochemical nanopipette liquid cells. Furthermore, the dynamic character of the dense phase offers a mechanistic basis for two key biological features of LLPS: its regulatory capacity in crowded cellular milieus and the facilitation of material exchange across condensate interfaces.

## Supplementary Material

nwag107_Supplemental_Files
