# Supplementary material for: Visualization of oligomerization, clustering, and density transition of intrinsically disordered proteins
Source: Natl Sci Rev. 2026 Feb 14;13(7):nwag107. doi: 10.1093/nsr/nwag107 (PMC13114874; doi:10.1093/nsr/nwag107)
Supplement: nwag107_Supplemental_Files [file nwag107_supplemental_files.zip › NSR_SI-2025-2345.R1-clean version-20260211.pdf]

Supplementary Information
for

Visualization of Oligomerization, Clustering, and Density Transition of Intrinsically Disordered Proteins

Jia-Ye Li[1], Yi-Hao Niu[1], Yi-Qin Gao[1], Huan Wang[1,2,3*]

[1] Beijing National Laboratory for Molecular Sciences, College of Chemistry and Molecular Engineering, Key Laboratory of Polymer Chemistry & Physics of Ministry of Education, Peking University, Beijing 100871, China;

[2] Beijing Advanced Center of RNA Biology (BEACON), Peking University; Beijing 100871, China;

[3] National Biomedical Imaging Center, Peking University, Beijing 100871, China

*Corresponding author: Huan Wang, wanghuan_ccme@pku.edu.cn

This PDF file includes:

Supporting text 1–11
Supporting Figure 1–5
Captions for Supporting Movie S1–10
SI References

Other supplementary materials for this manuscript include the following:

Movie S1 to S10
Molecular Dynamics (MD) Simulation Results

1. Materials

Quantifoil@R 1.2/1.3 gold grids with a mesh size of 300 were purchased from Beijing EBO Tech. Ltd. The 2-layer graphene grown by chemical vapor deposition (CVD) on copper was bought from ACS Material (Pasadena, CA, USA), while ammonium persulfate was obtained from Sigma-Aldrich. Milli-Q IQ7000 (Merk, Shanghai, China) produced the H_2O with a resistivity of 18.2 MΩ·cm.

FUS-LCD was purchased from Sangon Biotech (Shanghai) Co., Ltd. Wuhan Branch. Buffer solution is composed of 20 mM HEPES (pH = 7.4, purchased from HARVEYBIO) and 50–250 mM as well as 1M NaCl (purchased from Aladdin); 20 mM MOPS (pH = 7.4, purchased from Beyotime), 150 mM KCl (purchased from Shanghai Macklin Biochemical Co., Ltd), and 5 mM $CaCl_2$ (purchased from BBI) or 20 mM Tris-HCl (pH = 7.4, purchased from Beyotime) and 100 mM NaCl (purchased from Aladdin). The sample solution was prepared by reconstituting lyophilized FUS-LCD powder. The concentration of FUS-LCD was estimated to be approximately 6–60 μM, above c_{sat}, [1] based on the molecular weight of the reconstituted lyophilized powder and the molecular weight of FUS-LCD.

Full-length human FUS (FL-FUS) was purchased from TargetMol (FUS Protein, Human, Recombinant, His & Myc). The protein was expressed in an *E.* coli system with an N-terminal 10×His tag and a C-terminal Myc tag; its predicted molecular weight is 60.9 kDa (UniProt P35637). The lyophilized powder was reconstituted in 20 mM HEPES (pH = 7.5) and 100 mM NaCl. The bulk concentration of FL-FUS was estimated to be approximately 4 μM, above c_{sat} (2 μM) [2] according to its molecular weight.

2 mg/ml (~ 44 μM, above c_{sat} (2 μM) [3]) Tau-441 (2N4R) was purchased from Stressmarq, and the buffer composition is 20 mM HEPES (pH = 7.5) and 10 mM NaCl.

2. Experimental procedures of LP-TEM

2.1 Preparation of GLCs

Graphene liquid cells (GLCs) were created by forming scrolls or creases when a graphene-covered TEM grid was flipped to make contact with a free-floating graphene sheet [4]. Commercially available CVD-grown graphene was etched using 0.1 M ammonium persulfate solution and subsequently transferred onto a holey carbon gold TEM grid. To clean the grids, they were immersed in water. Approximately 0.5 μL of the sample solution was then added to the center of the graphene side of the grid. This grid was carefully and quickly placed onto a pre-etched, floating two-layer graphene sheet, with the droplet side facing down. After placement, the grid was left undisturbed for about 5 minutes to allow the formation of the liquid cells [5].

2.2 TEM Instruments and Cameras

Our TEM experiments were performed using JEOL-2100 Plus HC TEM equipped with a Gatan Oneview IS camera (2×2 pixels^2 binning resulting 1024×1024 pixels^2) at 80 kV. Exposure time was set as 40 ms or 160 ms, and no lag between frames.

2.3 Imaging conditions and buffer conditions

Movie S1: Oligomers and small clusters in HEPES and Tris buffer.

Top: *In-situ* TEM movie shows the existence of oligomers and small clusters in HEPES. FUS-LCD was reconstituted in 20 mM HEPES (pH = 7.4), 150 mM NaCl and protein bulk concentration is ~30 μM, above c_{sat} (1 μM). The movie is recorded at 80 kV with an electron dose rate of 3.3 $e^{-}Å^{-2}s^{-1}$, the exposure time is 160 ms, and the scale bar is 100 nm. A five-frame rolling average was applied to the video to enhance visualization.

Bottom: *In-situ* TEM movie shows the existence of oligomers and small clusters in Tris-HCl. FUS-LCD was reconstituted in 20 mM Tris-HCl (pH = 7.4), 100 mM NaCl and protein bulk concentration is ~60 μM, above c_{sat} (1 μM). The movie is recorded at 80 kV with an electron dose rate of 4.8 $e^{-}Å^{-2}s^{-1}$, the exposure time is 160 ms, and the scale bar is 100 nm. A five-frame rolling average was applied to the video to enhance

visualization.

Movie S2: Clusters and small oligomers in a buffer solution containing 1 M NaCl

In-situ TEM imaging captures both large clusters and small oligomers in a buffer solution containing 1 M NaCl. FUS-LCD was reconstituted in 20 mM HEPES (pH = 7.4), 1 M NaCl and protein bulk concentration is ~30 μM, above c_{sat} (1 μM). The movie is recorded at 80 kV with an electron dose rate of 3.6 $e^{-}Å^{-2}s^{-1}$, the exposure time is 160 ms, and the scale bar is 100 nm. A five-frame rolling average was applied to the video to enhance visualization.

Movie S3: Dense phase in 40% glycerol (v/v) buffer.

In-situ TEM movie captures the existence of dense phase. FUS-LCD was reconstituted in 20 mM HEPES (pH = 7.4), 150 mM NaCl with 40% glycerol (v/v) and protein bulk concentration is ~18 μM, above c_{sat} (1 μM). The movie is recorded at 80 kV with an electron dose rate of 2.7 $e^{-}Å^{-2}s^{-1}$, the exposure time is 160 ms, and the scale bar is 100 nm.

Movie S4: Clusters form from oligomers.

In-situ TEM movie shows IDP-IDP interaction between FUS-LCD forming small clusters from trimers (marked with cyan, green, and pink circles), dimers (marked with orange and purple circles), and monomers (distinguished by their projection area for a single molecule and marked with a blue circle) of FUS-LCD corresponding to **Fig. 1a**. The upper left panel displays the raw TEM data, while the upper right panel highlights the FUS-LC monomers with colorful circles. The lower left panel presents the binarized images corresponding to the raw TEM data, and the lower right panel illustrates the trajectories of the oligomers. FUS-LCD was reconstituted in 25 mM MOPS (pH = 7.4), 150 mM KCl, and 5 mM $CaCl_2$ and protein bulk concentration is ~6 μM, above c_{sat} (1 μM). The movie is recorded at 80 kV with an electron dose rate of 7.0 $e^{-}Å^{-2}s^{-1}$, the exposure time is 160 ms, and the scale bar is 10 nm.

Movie S5: Density transitions and quantification in GLC2

This *in-situ* TEM movie captures four density transition processes in GLC2, exhibiting a similar density transition process as shown in **Fig. 1b**, recorded at 80 kV. The movie plots the time-dependent changes in projected area, intensity, roundness, and the ratio of projected area to contour area. The colors blue, pink, purple, and orange correspond to density transition processes 1 to 4, respectively. FUS-LCD was reconstituted in 20 mM HEPES (pH = 7.4) and 250 mM NaCl and protein bulk concentration is ~30 μM, above c_{sat} (1 μM). The local protein concentrations in the dense phases are 36 mM, 92 mM, 109 mM, and 111 mM, corresponding to density transition processes 1 through 4, respectively. The electron dose rate is 11.2 $e^{-}Å^{-2}s^{-1}$, the exposure time is 40 ms, and the scale bar is 10 nm.

Movie S6: Density transitions and quantification in GLC3

In-situ TEM movie shows density transitions in GLC3. The movie plots the time-dependent changes in projected area, intensity, roundness, and the ratio of projected area to contour area. The colors blue, pink, purple, orange, and yellow correspond to density transition processes 5 to 9, respectively. FUS-LCD was reconstituted in 20 mM HEPES (pH = 7.4) and 50 mM NaCl and protein bulk concentration is ~30 μM, above c_{sat} (1 μM). The local protein concentrations in the dense phases are 109 mM, 23 mM, 47 mM, 76 mM and 19 mM, corresponding to density transition processes 5 to 9 respectively. The movie is recorded at 80 kV with an electron dose rate of 5.6 $e^{-}Å^{-2}s^{-1}$, the exposure time is 160 ms, and the scale bar is 10 nm.

Movie S7: Unstable Clusters ($c < c_{sat}$) *versus* Reversible Dense Phase ($c > c_{sat}$)

Left: The *in-situ* TEM movie demonstrates the existence and disassembly of clusters in a diluted FUS-LCD concentration ($c < c_{sat}$). A network-like structure, similar to those in **Movies 5 and 6**, is present in this movie and is highlighted by a yellow square. The time-dependent changes in projected area, intensity, roundness, and the ratio of projected area to contour area of this network-like structure are plotted in the movie. After some time, it disperses into several smaller aggregates, highlighted by red squares, and subsequently evolves into larger clusters, highlighted by blue squares. FUS-LCD was reconstituted in 20 mM HEPES (pH = 7.4) and 150 mM NaCl and protein bulk

concentration is ~ 600 nM, below c_{sat} (1 μM). FUS-LCD was diluted by 100-fold. The movie is recorded at 80 kV, 2.6 $e^{-}Å^{-2}s^{-1}$, the exposure time is 160 ms. Scale bar: 50 nm.

Right: *In-situ* TEM movie shows density transitions of larger FUS-LCD aggregates ($c > c_{sat}$). Despite being composed of closely packed molecules, the dense phase is highly dynamic and can repeatedly disassemble and reassemble at other locations corresponding to **Fig. 1f**. The movie plots the time-dependent changes in projected area, intensity, roundness, and the ratio of projected area to contour area. FUS-LCD was reconstituted in 20 mM HEPES (pH = 7.4) and 150 mM NaCl and protein bulk concentration is ~30 μM, above c_{sat} (1 μM). The movie is recorded at 80 kV, 4.2 $e^{-}Å^{-2}s^{-1}$, the exposure time is 160 ms. Scale bar: 20 nm.

Movie S8: Fusion of two dense phases.

In-situ TEM movie shows growth and fusion of two dense phases (highlighted be yellow box). FUS-LCD was reconstituted in 20 mM HEPES (pH = 7.4), 150 mM NaCl and protein bulk concentration is ~30 μM, above c_{sat} (1 μM). The movie is recorded at 80 kV with an electron dose rate of 1.5 $e^{-}Å^{-2}s^{-1}$, the exposure time is 160 ms, and the scale bar is 100 nm. A five-frame rolling average was applied to the video to enhance visualization.

Movie S9: Coexistence of all forms: monomer, oligomers, cluster and dense phase.

In-situ TEM movie captures dense phase (outlined by a yellow box, 1–9 s) disassembles and reassembles, yielding several smaller dense phases (blue box, after 10 s) and oligomers (blue arrows within the blue box), with clusters (purple box) coexisting. FUS-LCD was reconstituted in 20 mM HEPES (pH = 7.4), 150 mM NaCl and protein bulk concentration is ~30 μM, above c_{sat} (1 μM). The movie is recorded at 80 kV with an electron dose rate of 2.6 $e^{-}Å^{-2}s^{-1}$, the exposure time is 160 ms, and the scale bar is 100 nm. A five-frame rolling average was applied to the video to enhance visualization.

Movie S10: General Pattern of Density Transitions observed in other IDPs.

Top: The *in-situ* TEM movie captures the existence and reassembly of the dense phase for Full Length FUS Protein (FL-FUS) in 20 mM HEPES (pH = 7.5) and 100 mM NaCl

and protein bulk concentration is ~4 μM, above c_{sat} (2 μM). The movie is recorded at 80 kV, 6.8 e^{-}Å^{-2}s^{-1}. Scale bar: 100 nm.

Bottom: The *in-situ* TEM movie visualizes the formation and dynamic reassembly of the dense phase for Tau protein in 20 mM HEPES (pH = 7.5) and 10 mM NaCl and protein bulk concentration is ~44 μM, above c_{sat} (2 μM). The movie is recorded at 80 kV, 2.6 e^{-}Å^{-2}s^{-1}. Scale bar: 100 nm.

2.4 Image processing

Time-series LP-TEM images in .dm4 format were converted to .png format using custom Python scripts. Drift correction was carried out using the "template-matching" plugin in ImageJ, with an immobilized feature chosen as a reference point. To enhance visual clarity, a small sub-region containing the region of interest (ROI) was cropped from each image. Subsequently, a rolling average custom Python scripts was applied to every set of five images to improve contrast. Binarized images were then generated using the UNet++ algorithm applied to the averaged images [6].

3 Technical details of LP-TEM

3.1 Electron beam effect

Electron beam effect in LP-TEM can be summarized as follows [7]: when electrons are incident on a sample, they can be categorized into elastically scattered electrons and inelastically scattered electrons. Elastically scattered electrons result from elastic collisions between the incident electrons and the sample atoms. The energy and momentum of the system composed of the incident electrons and the sample atoms are conserved. However, when the energy transferred by the electron in an elastic collision exceeds the threshold energy for atomic displacement, it can cause atomic displacement or the release of atoms from the sample surface as free atoms, resulting in knock-on damage. Inelastically scattered electrons arise from collisions between the incident electrons and the extranuclear electrons of the sample atoms. Most of the energy lost by the incident electrons is converted into heat, typically in the range of 5–100 eV (with an average of 20 eV) [8] which can excite the valence electrons involved in forming covalent bonds outside the atomic nucleus, leading to the breaking of chemical bonds, the generation of free radicals and secondary electrons, and causing primary radiation damage. The electrons released during the primary damage process can continue to disrupt more chemical bonds, while free radicals can initiate a series of chemical reactions, resulting in secondary damage. In LP-TEM, the interaction between incident electrons and the solvent (typically deionized water) leads to water radiolysis [9], producing a series of radiolysis products such as free radicals, solvated electrons, and hydrogen gas [10, 11]. It has been reported that in the range of electron accelerating voltages used for microscopy, radiolysis is often the more dominant phenomenon [12, 13]. Admittedly, the electron beam effect has always been one of the greatest concerns when it comes to imaging biological samples using LP-TEM. Almost every article in this field evaluates the impact of electron dose, with comprehensive reviews detailing the considerations necessary for imaging biological samples using LP-TEM. This includes assessments of safe electron doses, the influence of chemical environments in liquid cells, and image signal-to-noise ratio. [14-19] In summary, whether due to knock-

on damage leading to chemical bond breakage, structural damage to biological macromolecules, or changes in the chemical environment resulting from radiolysis of water, all are closely linked to electron dose. When the electron beam irradiates the liquid cell, interactions with the window materials of the liquid cell led to charge accumulation and knock-on damage of the window material. Interactions with the liquid result in radiolysis, altering the chemical environment within the liquid cell. Interaction with biological molecules leads to changes in their structure and function. Yet, Numerous research and review articles have discussed, evaluated and proposed possible solutions to the damage caused by electron beam [9, 20-24].

For the GLC-based LP-TEM used in this study, graphene has high thermal [25] and electrical conductivities [26], so the effects of beam-induced sample heating and charge accumulation are reduced. In addition, graphene can act as a radical scavenger, mitigating the impact of radiolysis-generated radicals in water under electron irradiation [27]. Thus, as a window material, graphene provides excellent protection for the encapsulated liquid sample, and the first consideration during LP-TEM imaging should be knock-on damage to the graphene window. Although a higher accelerating voltage reduces the electron–sample interaction cross-section, meaning the sample absorbs less radiation for the same electron dose, the increased incident electron energy can exceed the knock-on damage threshold of graphene (60 and 80 keV) [28], displacing carbon atoms and compromising graphene's encapsulation and protective functions. Using cryo-EM as a reference, damage to biological samples in cryo-EM mainly arises from bond cleavage, radical formation, and the evolution of secondary gases. In liquids, radiolysis products such as radicals can diffuse rapidly, participate in subsequent reactions, and be consumed; their effective reactivity is also lower than under low-temperature conditions. Combined with graphene's radical-scavenging effect, the "safe" electron dose that samples can tolerate in LP-TEM (cumulative dose 500–1000 $e^{-}Å^{-2}s^{-1}$) [13, 22, 23] should be 1–2 orders of magnitude higher than in cryo-EM (about 100 $e^{-}Å^{-2}s^{-1}$ for imaging protein structures and about 10 $e^{-}Å^{-2}s^{-1}$ for imaging active enzymes). This estimate is consistent with current LP-TEM experiments imaging

dose-sensitive biological samples: at dose rates of 2–10 $e^{-}Å^{-2}s^{-1}$. Granick *et al.* observed the entire DNA hybridization process and captured intermediates [29], as well as chain elongation of primers and monomers catalyzed by DNA polymerase [5], demonstrating that electron-beam-sensitive biomolecules can retain structural and functional integrity under "safe" electron doses. In summary, electron–sample interactions are the basis of LP-TEM imaging; graphene as a window material affords maximal protection to the encapsulated liquid sample. By appropriately choosing the accelerating voltage and the electron dose rate during imaging, one can achieve at least ~100 s of continuous imaging while preserving the structural and functional integrity of biomolecules.

Moreover, it has been experimentally demonstrated that the electron-dose tolerance of radiation-sensitive tubulin encapsulated in graphene is an order of magnitude higher than that of microtubules vitrified in amorphous ice; their protofilament features remain preserved even at dose rates as high as $(7.2 \pm 1.4) \times 10^2$ $e^{-}Å^{-2}s^{-1}$ [30]. Gianneschi *et al.* [31] employed MALDI-imaging mass spectrometry (MALDI-IMS) as a post-mortem analysis after LP-TEM imaging technique. The intact peptide signal by MALDI-IMS confirmed the integrity of peptide imaging at an electron dose rate of $0.11 \pm 0.07\, e^{-}\, Å^{-2}\, s^{-1}$ in SiN_x liquid cell and determined an accumulated electron dose of 10^2–10^3 e^{-} $Å^{-2}$, which varied depending on the structural differences in peptide segments. Furthermore, the liquid environment promotes rapid diffusion of radicals, markedly enhancing the role of graphene as a radical scavenger in LP-TEM and mitigating beam-induced water radiolysis, thereby increasing the tolerance of amyloid fibrils to cumulative electron dose by about 40-fold [32]. In addition to graphene, the incorporation of D_2O further increases the tolerable dose via isotope effects [33]. Consequently, biomacromolecules imaged by LP-TEM in GLCs can undergo several minutes of non-destructive imaging, facilitating exploration of all possible conformational states in their native liquid environment and enabling imaging of individual proteins with delicate secondary and tertiary structures. As research has progressed, concerns about using LP-TEM to track dynamic processes of biological specimens in solution have gradually diminished. Moreover, because

enzyme activity depends strongly on secondary structure and an intact three-dimensional architecture [34], observing preserved enzymatic activity performing its physiological function during an LP-TEM experiment would confirm that, despite electron-beam exposure, surface interactions, and radiolysis, bio-macromolecular structure and function remain intact within the imaging window and reflect conformational dynamics consistent with those in bulk solution. For example, LP-TEM experiments have observed single-stranded DNA undergoing complementary base pairing in accordance with its normal physiological function [29], as well as DNA polymerase catalyzing DNA chain elongation [5], indicating that biomacromolecules such as DNA and proteins can retain structural and functional integrity at commonly used LP-TEM electron dose rates (2–10 $e^{-}Å^{-2}s^{-1}$). In summary, the evidence supporting the viability of imaging biological samples using LP-TEM can be classified into the following three aspects: First, in LP-TEM experiments, the structure of biological macromolecules remains intact [30, 31]; Second, functions of biomacromolecules preserve under LP-TEM imaging conditions [5, 29, 33]. Thirdly, biological macromolecules maintain the same conformational distribution as in bulk solution [35].

In this work, we rigorously controlled the electron dose rate, keeping it below 10 $e^{-}Å^{-2}s^{-1}$ to minimize beam effects. We do not attribute the observed density transition to the electron beam, because, in general, before any density transition occurs, FUS-LCD molecules (indicated be yellow arrows at 0 s in **Fig. S1a**) are damaged due to radiolysis effect and rapidly disappear at 90 s as shown in **Fig. S1a**; within the next few seconds, continued radiolysis generates bubbles that occupy the GLCs as shown at 94 s in **Fig. S1a** where the air-liquid interface is indicated by the red arrow and red dashed line. These signatures indicate beam damage that would preclude, rather than induce, the transition. After the density transition is complete, the dense phase will also most likely disappear due to radiolysis as shown in 21 s in **Fig. S1b**. Moreover, the observed disassembly–reassembly cycles are also unlikely to be beam-driven: they occur repeatedly and typically precede bubble formation caused by radiolysis, as shown in **Movie S10**.

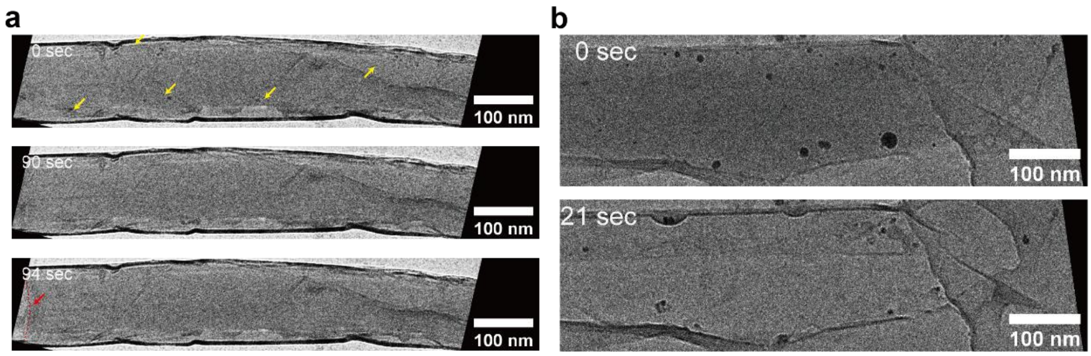

Figure S1. Disappearance of FUS-LCD molecules (a) and dense phase (b) caused by prolonged electron-beam irradiation. (a) FUS-LCD molecules (yellow arrows) are present in the GLC. Around 90 s, the oligomers disappear due to electron-beam effects. At 94 s, bubbles form in the liquid as a result of radiolysis, as indicated by the red arrows and red dashed lines. **(b)** The dense phase in the GLC disappears due to electron-beam effects. ~30 μM FUS-LCD was reconstituted in 20 mM HEPES (pH = 7.4) and 150 mM NaCl. The movie is recorded at 80 kV. The electron dose rate is 2.7 $e^{-}Å^{-2}s^{-1}$, the exposure time is 160 ms, and the scale bar is 100 nm.

3.2 Graphene substrate effect

In GLC, molecules are confined to a liquid cell with tens to hundreds of nanometers thick between two graphene sheets [5, 36], where strong interfacial interactions suppress diffusion by up to roughly eight orders of magnitude—an effect observed for both nanoparticles [21, 37], short DNA oligomers [29] and lipid–protein dynamic [13]. In other words, if the motion were normal Brownian diffusion, then based on the molecule's Stokes–Einstein diffusion coefficient in water, we would not be able to image it clearly with a 160 ms exposure time. Yet in LP-TEM we can clearly analyze biomolecules, indicating that their motion is significantly slowed. The possible mechanisms are enhanced interfacial friction [38-40] and, more importantly, transient adsorption/desorption to the graphene surfaces [5], which intermittently immobilizes molecules yet still allows flexible molecules to sample conformations similar to those in free solution. Meanwhile, electron-beam effects can further modulate the kinetics. [35]. As a result, processes that would occur on microsecond timescales in bulk solution are stretched to seconds or longer in a GLC. Accordingly, near the

adsorption–desorption transition, GLC based LP-TEM reports equilibrated, time-averaged conformational distributions from a few molecules, effectively functioning as a slow-motion camera [35].

3.3 Protein Concentration in GLC

In GLCs, each GLC stochastically encapsulates a certain volume of solution (a typical GLC is tens to a few hundred nanometers wide, hundreds of nanometers to several micrometers long, and tens to several hundred nanometers thick) and an uncertain number of molecules during formation [5, 36]. As a result, the concentration inside an individual GLC can deviate to some extent from the initial bulk concentration. To ensure consistency in discussing concentration effects, throughout the main text we report the solution concentration as the initial bulk concentration, which may be either above (6–60 μM) or below (600 nM) the saturation concentration.

4. Consistency of density phase among different buffer conditions

Prior studies have extensively examined the effects of salt concentration, buffer viscosity, and bulk protein concentration [41-44]. Among these, bulk protein concentration was the primary determinant of whether the transition occurred in our work, whereas the transition remained reproducible across variations in salt and viscosity. First, in our experiments, the choice of buffer (MOPS, HEPES, or Tris) did not affect the appearance of oligomers, clusters, or the dense phase as shown in **Fig. S2, Movies S1** and **S4** since MOPS, HEPES and Tris are all widely used biological buffers. Moreover, we observed the similar behavior across salt concentrations crossing the physiological range (50–250 mM NaCl) in **Movies S5–7** and extending beyond it (1 M NaCl) as shown in **Movie S2**. We also increased solution viscosity by adding 40% (v/v) glycerol and still observed the coexistence of clusters and dense phase as shown in **Movie S3**. Together, these results indicate that, at early stages of LLPS and within the buffer systems used here, multistep nucleation is prevalent and insensitive to buffer composition. Nevertheless, the effects of salt concentration and viscosity on the kinetics of multistep nucleation remain to be quantified in future work.

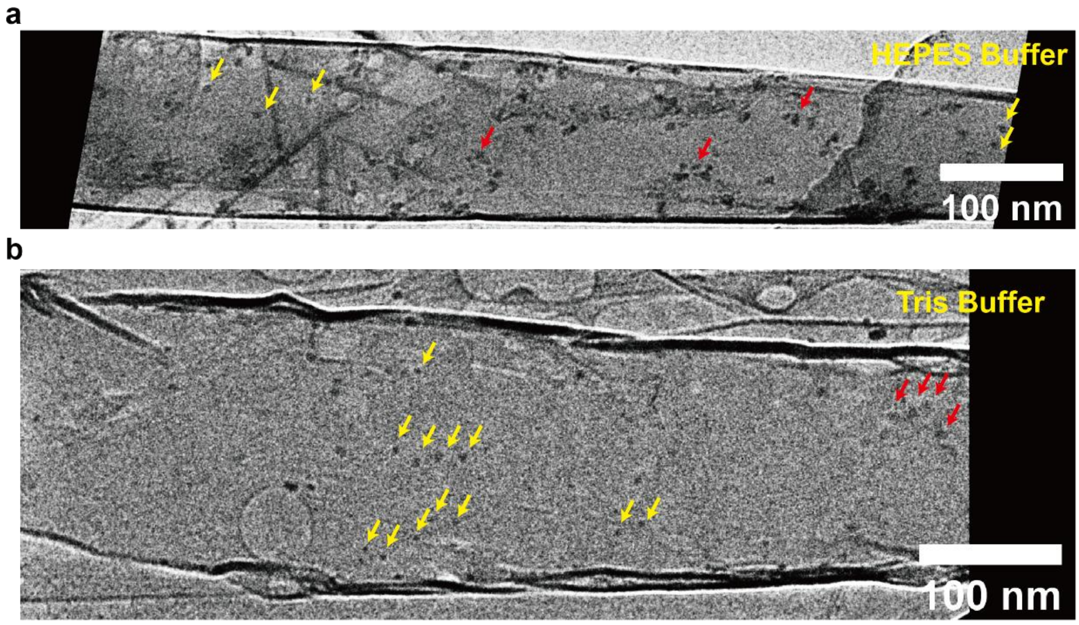

Figure S2. FUS-LCD molecules in HEPES buffer (a) and Tris-HCl buffer (b) corresponding to Movie S1. Yellow arrows indicate representative FUS-LCD

molecules with a single distinguishable subunit (projected area corresponding to one subunit). Red arrows indicate representative oligomers composed of several distinguishable subunits, with projected areas approximately integer multiples of that of a monomer. Scale bar: 100 nm.

5. Molecular Dynamics Simulation Methods

5.1 System Preparation and Modeling

The initial structure for molecular dynamics (MD) simulations was obtained from the AlphaFold2-predicted monomeric structure of the human FUS protein (UniProt ID: P35637) [45]. The structure was protonated using the H++ web server [46] with the following parameters: an external dielectric constant of 80, an internal dielectric constant of 10, a salt concentration of 0.15 M, and a target pH of 7.4.

All MD systems were constructed using the **tleap** module of the **Amber24** software package [47]. The protein was modeled using the **ff19SB** force field [48], while water molecules were represented by the **OPC four-point water model** [49]. The protonated protein was placed at the center of a rectangular water box, with a minimum distance of 10.0 Å between the protein surface and the box boundaries.

Simulation system with 100 mM NaCl was prepared: First, Na^+ ions were randomly added using the 'addIonsRand' command to neutralize the net charge of the system. Additional Na^+ and Cl^- ions were then randomly introduced to achieve the desired salt concentration. All systems were electrically neutral.

5.2 Molecular Dynamics Simulation Protocol

All MD simulations were performed using the GPU-accelerated **pmemd.cuda** module in **Amber24**.

Prior to production simulations, each system underwent a two-stage energy minimization to remove unfavorable atomic contacts. In the first stage, all protein heavy atoms were restrained with a force constant of 100 $kcal \cdot mol^{-1} \cdot Å^{-2}$ to relax solvent and ions. This minimization consisted of an initial steepest descent phase followed by conjugate gradient optimization, for a total of 1000 steps. In the second stage, weaker restraints (10 $kcal \cdot mol^{-1} \cdot Å^{-2}$) were applied only to protein backbone atoms (Cα, N, and C), and another 1000 steps of minimization were performed to allow further side-chain relaxation. During both minimization stages, all bonds involving hydrogen atoms were constrained using the SHAKE algorithm, and a nonbonded interaction cutoff of 10.0 Å

was applied.

The system was then gradually heated under the NVT ensemble from 100 K to 298 K over 1 ns, corresponding to 1,000,000 steps with a 1 fs time step. During heating, positional restraints (100 $kcal \cdot mol^{-1} \cdot Å^{-2}$) were applied to all protein heavy atoms.

Following heating, the system was equilibrated under the NPT ensemble for 2 ns at 298 K and 1 bar. Strong positional restraints (100 $kcal \cdot mol^{-1} \cdot Å^{-2}$) on protein heavy atoms were maintained during the first 1 ns and then reduced to 10 $kcal \cdot mol^{-1} \cdot Å^{-2}$ during the second 1 ns.

A subsequent 5 ns staged restraint-release equilibration was conducted, during which only protein backbone atoms were restrained. The restraint force constants were gradually reduced as follows: 10 $kcal \cdot mol^{-1} \cdot Å^{-2}$ for the first 2 ns, 1 $kcal \cdot mol^{-1} \cdot Å^{-2}$ for the next 1 ns, 0.1 $kcal \cdot mol^{-1} \cdot Å^{-2}$ for the following 1 ns, and finally removed entirely during the last 1 ns. This stage was carried out under the NPT ensemble with a 1 fs time step,.

Production MD simulations were then performed without any positional restraints for **100 ns**. All production runs were conducted under the NPT ensemble at 298 K and 1 bar. Bonds involving hydrogen atoms were constrained using SHAKE, allowing a time step of **2 fs**.

5.3 Computational Resources

All molecular dynamics simulations were carried out on high-performance computing nodes equipped with **NVIDIA GeForce RTX 4090 GPUs**. Each simulation was allocated one GPU and 16 GB of system memory.

6. Quantification from LP-TEM images

6.1 Calculation of R_g^{2D}

In order to quantify the size of protein molecules, we calculated radius of gyration for each molecule from the two-dimensional projection images using custom Python code [50]. The original definition of the radius of gyration is:

$$R_g^{3D} = \sqrt{\frac{1}{M}\sum_{i}^{N} m_i(r_i - \bar{r})^2}$$

where $M = \sum_i^N m_i$ is the total mass of the molecule, *N* is the total number of atoms, and r_i is the position vector of the ith atom relative to the center of mass. The mass-weighted centroid is thus:

$$\bar{r} = \frac{1}{M}\sum_{i=1}^{N} m_i r_i$$

In LP-TEM experiments, the LP-TEM images we analyze are two-dimensional projections of protein molecules rather than their full three-dimensional structures, so the original formula needs to be adapted to the characteristics of 2D LP-TEM images. In this way, we first obtain a binarized image consisting only of black and white pixels; the white region corresponds to the target molecule, and the black region corresponds to the background. Using the white region, we compute the geometric center of the target molecule, which can be approximated as the centroid of the protein molecule in its 2D projection. Next, we determine the coordinates of every pixel within the white region of the binary image; these pixels represent the area occupied by the protein molecule in the 2D projection. We then measure the distance from each pixel to the centroid, denoted r_i. To account for the influence of pixel grayscale values, we weight these distances by the grayscale value g at each point in the EM image and use the following formula to compute the radius of gyration R_g^{2D}:

$$R_g^{2D} = \sqrt{\frac{\sum g \times r_i^*}{\sum g}}$$

corresponding to the 2D LP-TEM image. Although R_g^{2D} is computed from a 2D projection and thus differs from the three-dimensional radius of gyration, it still provides important information about the morphology of proteins, especially when comparing size differences between different conformations or different molecules. By combining the projected area and the radius of gyration R_g^{2D}, we can gain a more comprehensive understanding of the structural features of protein molecules.

In order to quantify and validate it as monomer, we compared the estimated R_g^{2D} with values reported in the literature from independent measurements and calculations [1] and found our $R_g^{2D} \approx 1.8$ nm (as shown in **Fig. S3a**) was much smaller than the SAXS data. We attribute this to several factors. First, projecting a three-dimensional object into two dimensions inevitably underestimates R_g^{2D} derived from 2D images. Because the molecules are not spherical, orientations in which the long axis is perpendicular to the xy plane further reduce R_g^{2D} in projection. Second, in our calculations we used pixel intensity as a surrogate for atomic mass, which introduces substantial error. This is exacerbated by overlap of atoms along the projection (z) direction, the fact that the TEM contrast–mass–thickness relationship may be non-linear, and the influence of the liquid layer, graphene, and the carbon support film on pixel grayscale. Third, even for a random coil, the radial segment-density distribution is non-uniform, becoming progressively sparser from the center outward. [51] Fourth, the R_g^{3D} measured by SAXS is an ensemble average; owing to molecular heterogeneity, R_g^{3D} should span a distribution rather than being concentrated near ~5 nm. Finally, the molecules we observed exhibit intermolecular interactions; the presence of neighboring molecules likely stabilizes transient secondary and even tertiary structures in IDPs, yielding an R_g^{2D} that differs from that of the random coils measured by SAXS. Consequently, we performed molecular dynamics (MD) simulations of the FUS low-complexity domain (FUS-LCD) and have provided methodological details in the

Supplementary Information. Using the MD trajectories and the MDTraj Python package, we computed the distribution of R_g^{3D}, which spans 2.66–5.23 nm. Because 2D projection underestimates R_g^{3D}, we barely considered the isotropic (random-orientation) average, for which $R_g^{3D} = \sqrt{\frac{3}{2}} R_g^{2D}$. Accordingly, the expected range of R_g^{2D} is 2.18–4.29 nm. On this basis, we classified particles whose sizes matched the MD results as monomers and then computed their projected areas by multiplying the per-pixel area by the number of white pixels in the binarized images.

6.2 Projected area, intensity, roundness, and the projected area-to-contour area ratio

Using binarized images as masks, the contour of each ROI was extracted with 'cv2.findContours' in custom Python scripts. These contours were then overlaid on the TEM images in different colors using 'cv2.drawContours'. The projected area was calculated by multiplying the pixel size by the number of white pixels in the binarized images. The intensity of each frame was calculated by averaging the gray value of each pixel within the white region in binarized images. The roundness of each frame was determined by first finding the merged contour using 'cv2.convexHull (all_points)', and then calculating the merged circularity with the formula: $Roundess = 4 \times \pi \times \frac{Area}{Perimeter^2}$. All contour points were pooled and their convex hull was computed to define a single "merged contour" as illustrated in the yellow circles on binarized images in **Fig. 1b.** We then quantified this merged contour by calculating its enclosed area as the contour area. In this way, the projected area-to-contour area ratio reflects molecular packing within the cluster. Ratios deviating from 1 indicate looser packing with larger gaps, while ratios near 1 suggest tight packing with minimal gaps.

7. Criteria of monomer, dimers, trimers, clusters and dense phase

Based on MD results, we classified particles whose sizes matched the MD results as monomers and then computed their projected areas by multiplying the per-pixel area by the number of white pixels in the binarized images. The projected area of single FUS-LCD molecules (~11.5 nm²) was calculated by averaging measurements from 1124 LP-TEM image frames of six molecules in **Fig. 1a** and **Movie S4**. The projected area was calculated by multiplying the pixel size by the number of white pixels in the binarized images.

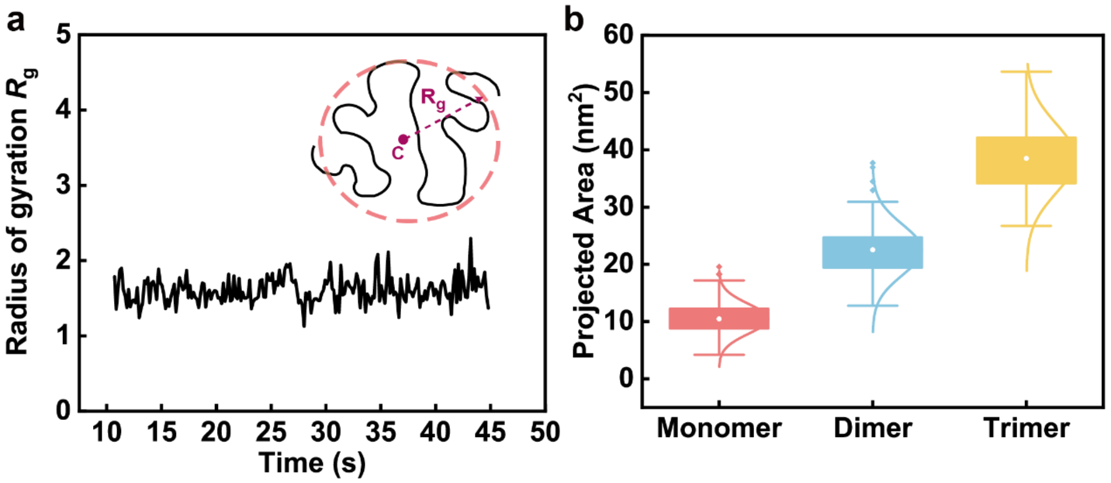

Figure S3. Quantification and identification of trimers, dimers, and monomers. (a) Time dependent changes on radius of gyration (R_g^{2D}) for an FUS-LCD monomer in **Movie S4**. Inset: schematic illustrating the calculation of R_g^{2D} for FUS-LCD. **(b)** Projected area distributions of trimers, dimers, and monomers observed in **Movie S4.**

For **Fig. 1a**, our identification procedure for dimers and trimers was as follows: in the LP-TEM images, dimers exhibit two distinguishable subunits and trimers exhibit three distinguishable subunits. We then calculated the projected areas of these regions. As shown in **Fig. S3b**, the projected areas of dimers and trimers are approximately twice and three times that of monomers, respectively. These findings establish the criteria used in **Fig. 1a** to identify trimers, dimers, and monomers. The term "oligomers" collectively refers to dimers and trimers; further aggregation of these species yields

clusters.

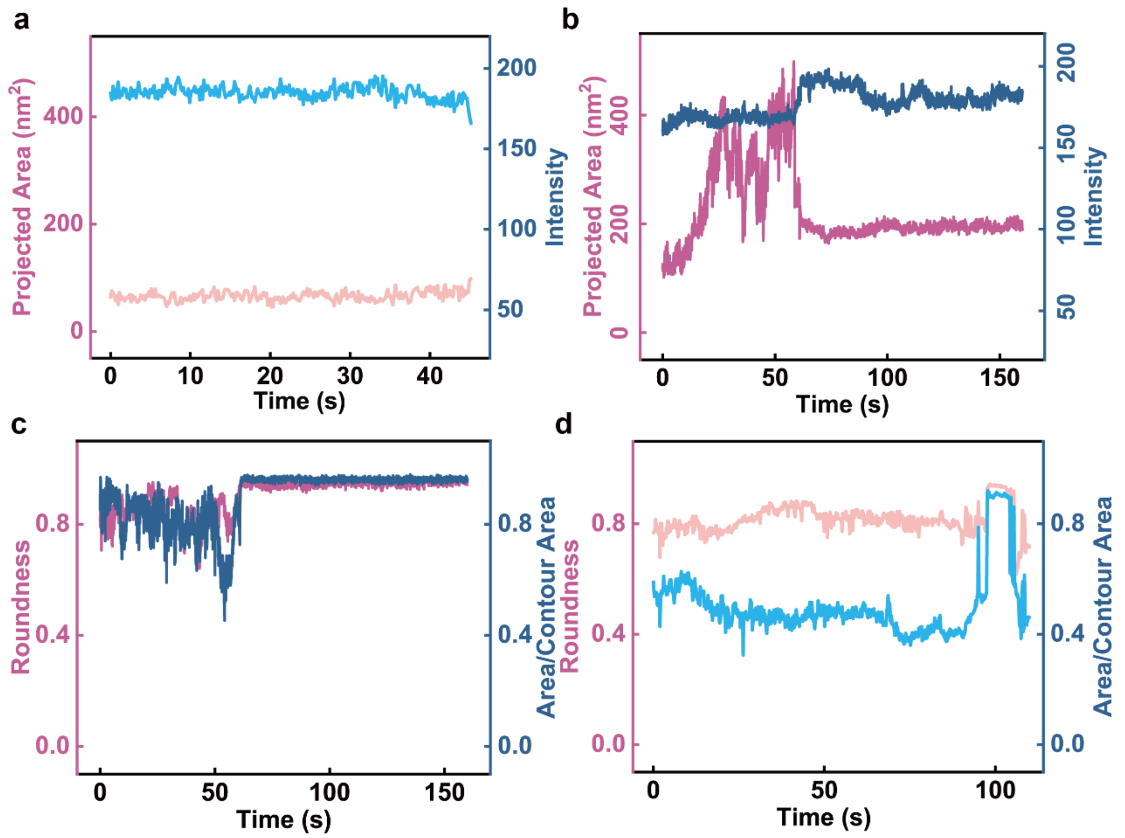

Figure S4. Quantification of density transition process. (a) Time-dependent changes in projected area (pink line) and intensity (light blue line) corresponding to oligomers in **Fig. 1a. (b)** Time-dependent changes in projected area (purple line) and intensity (dark blue line) corresponding to clusters and dense phase in **Fig. 1b. (c)** Time-dependent changes in roundness (purple line) and projected area-to-contour area ratio **(**dark blue line**)** corresponding to oligomers clusters and dense phase in **Fig. 1b.** Time-dependent changes in roundness (purple line) and projected area-to-contour area ratio **(**dark blue line**)** corresponding to oligomers clusters and dense phase in **Fig. 1b. (d)** Time-dependent changes in roundness (pink line) and projected area-to-contour area ratio (light blue line) corresponding to dissemble of dense phase in **Fig. 1f.**

Then, we delineate clusters and dense phases primarily by tracking changes in four quantitative metrics: projected area, intensity, roundness, and the projected area-to-contour area ratio. We define clusters as network-like structures in LP-TEM images with irregular outer boundaries, composed of interconnected small regions and

exhibiting lower contrast than the dense phase; they represent the stage prior to the density transition. In contrast, the dense phase is the state after the density transition, characterized by an approximately circular shape with a highly compact and extremely dense interior.

The specific quantitative criteria are: First, we define the area of the white regions in the binarized images of **Fig. 1a–b** and **f** as the projection area for each frame. We also identify the outermost boundary encompassing all particles and define the area within the periphery boundary as the contour area as shown in the binarized images identified by yellow circle in binary images shown in **Fig. 1a–b** and **f**. Compared to the cluster formation process shown in **Fig. 1a**, where projected area (light pink line in **Fig. S4a**) and intensity (light blue line in **Fig. S4a**) remain relatively stable, the density transition exhibits a sharp drop in projected area (purple line in **Fig. S4b**) and a significant increase in intensity (dark blue line in **Fig. S4b**). As shown in **Fig. 1b**, the network-like structure evolves from interconnected small regions into a nearly spherical dense area, accompanied by an increase in intensity (dark blue line in **Fig. S4b**) and roundness (purple line in **Fig. S4c**) and a decrease in projected area (purple line in **Fig. S4b**). We infer that the sudden increase/decrease point is the density transition point. Before this point we refer the structure as cluster and after this point we refer the structure as dense phase. Additionally, the projected area-to-contour area ratio (dark blue line in **Fig. S4c**) deviates from 1, indicating a loose arrangement of clusters within the outer contour (0–60 s in dark blue line in **Fig. S4c**, similar to 27.16 s in **Fig. 1b**), with larger gaps between clusters. Conversely, a ratio closer to 1 suggests almost no gaps between clusters, indicating tight packing (after ~60 s in dark blue line in **Fig. S4c**, similar to 62.08 s in **Fig. 1b**). Therefore, the projected area-to-contour area ratio reflects the compactness of the clusters forming the network. Considering that increased image intensity and cluster compactness indicate a higher number of molecules per unit area, we conclude that density transitions are characterized by the formation of a highly compacted circular dense phase.

8. Calculation and discussion on the second virial coefficient B_2'

Adapting a technique commonly used in light-scattering experiments, we estimated the second virial coefficient, B_2', from a Zimm plot [52]. Since the second virial coefficient B_2 reflects protein-protein interactions, we replaced light scattering intensity by LP-TEM image intensity (representing electron scattering) and replaced concentration with cluster projection area (corresponding to molecule number) in classical equation $\frac{Kc}{R(\theta)}=\frac{1}{M_w}+2B_2c$ in Zimm plot. Here, K is the light scattering constant, c is the solution concentration, $R(\theta)$ is the Rayleigh ratio, M_w is the weight-average molar mass and B_2 is the second virial coefficient.

B_2' is calculated as follows: First, assuming a constant K of 1.0, the target variable $\frac{Kc}{R(\theta)}$ was calculated by replacing $R(\theta)$ by LP-TEM image intensity and replacing c with projection area. The linear equation $\frac{Kc}{R(\theta)} = \frac{1}{M_w} + 2B_2c$ was defined, where M_w and B_2' were the fitting parameters. Initial guesses and reasonable bounds for the fitting parameters were set to ensure the stability of the fitting process. In the customized python code, the 'curve_fit' function from the 'scipy.optimize' library was used to perform nonlinear least squares fitting on the data, yielding the optimal fitting parameters M_w and B_2'. The molar mass M_w was calculated by taking the reciprocal of 'Mw_inv'. Special cases where 'Mw_inv' equals zero were handled appropriately. To evaluate the quality of the fit, residuals, the sum of squared residuals (ss_res), and the total sum of squares (ss_tot) were computed, and the R-squared value was derived. The results showed that the R-squared value was close to 1, indicating a good fit. The fitting parameters, their covariance matrix, the R-squared value, and the calculated molar mass M_w were stored in a new DataFrame. Subsequently, these results, along with the input data, were saved to a new Excel file for further analysis and verification. Finally, the 'matplotlib' library was used to plot the original data points and the fitted curve, and the image was saved as a PNG file. This image clearly illustrated the relationship between the data points and the fitted curve, further validating the effectiveness of the fitting process. Through these steps, this study successfully

achieved linear fitting of data extracted from an Excel file, analyzed the relationship between variables, and visually presented the results in graphical and tabular forms.

Temporal analysis of density transition videos (100-frame per segments) demonstrated a decrease in B_2' during the transition process (yellow pentagons from 80s to 96 s in **Fig. 1c**), indicating a reduction in intermolecular repulsive forces within the dense phase. Of the measured states, oligomers exhibited the highest normalized second virial coefficient (B_2' $\approx$ 0.18), a value approximately two orders of magnitude greater than that observed for clusters and dense phases, (1.9–3.5) $\times 10^{-3}$. This relatively large positive value indicates that these early-stage species are highly solvated and maintain an expanded state dominated by conformational disorder and charge repulsion, likely consistent with the thermodynamic behavior of proteins in good solvents [53]. Tracking the dynamic evolution during the density transition revealed a statistically significant decline in B_2' from 2.9×10^{-3} to 2.6×10^{-3} (64–80 s in **Fig. 1c**). This reduction reflects a fundamental attenuation of intermolecular repulsion, implicating conformational rearrangements—possibly involving de-solvation and structural compaction—as the key driver facilitating the transition into the condensed phase [54].

***9. Calculation of molecule number* N and c_{dense}**

The number of molecules (*N*) within a cluster was estimated by dividing the projected area before the abrupt area contraction during the density transition by the calculated single-molecule area. The dense phase concentration c_{dense} was determined from the highly compacted region after the density transition. Specifically, the radius of the dense phase was measured using ImageJ, and the volume was calculated assuming a spherical geometry using $V = \frac{4}{3}\pi R^3$. The concentration $c = \frac{n}{V}$ was then calculated using the estimated number of molecules N ($n = \frac{N}{6.02\times10^{23}}$) and the calculated volume V.

10. The dense phase is dynamic and reversible

After a dense phase forms, it can grow either by the addition of oligomers/clusters or by fusion of two droplets (**Fig. S5a** and **Movie S8**). In addition, we also observed that the dense phase can spontaneously dissolve into smaller oligomers or individual molecules, which later reassemble into a dense phase. Apart from this, the dense phase is highly dynamic and reversible as shown in **Fig. 1f** as well as **Movies S7 and S9**. Although tightly packed and concentrated with stronger intermolecular interaction, IDP within this phase allow for continuous reorganization and movement of molecules. Dense phase exhibits a dynamic and reversible nature. Over time, the dense phase can spontaneously disassemble into smaller oligomers or individual molecules, which can later reassemble into a dense phase again.

Disassembly of the dense phase can lead to the coexistence of clusters and dense condensates, as shown in **Fig. S5b** and **Movie S9**. In **Fig. S5b**, at 0 s, the dense phase (yellow dashed box) begins to disassemble; by ~8 s, it has reassembled into smaller condensates and oligomers (blue dashed boxes and arrows). During this interval, dense phases, oligomers, and clusters (purple dashed boxes) coexist. This behavior likely reflects two coupled factors. First, in GLC the total protein content is finite, unlike in bulk solution; as a droplet grows, it depletes the surrounding dilute phase, and the resulting drop in outside concentration — together with the extremely low interfacial tension of IDP condensates [55] — can reduce supersaturation sufficiently to reverse growth and drive shrinkage and dissolution. Once local monomer levels are replenished, the same region can readily re-nucleate a new dense phase. Second, the "dense phase" here refers to nanoscopic, metastable condensates (radii of a few tens of nanometers) rather than macroscopic, fully equilibrated droplets; even micron-scale droplets are known to exchange material rapidly with the surrounding phase and to partition components selectively. Although experimental factors (e.g., electron-beam–induced local chemical changes, interfacial adsorption to graphene) may modulate the kinetics,

we observe similar dissolution–reassembly cycles across multiple GLCs as shown in **Movies S7–10** indicating that the reversibility arises intrinsically from weak multivalent interactions coupled to finite-size thermodynamics. In summary, the dense phase can disassemble and later reassemble refers to nanoscopic, near-saturation, metastable condensates that undergo spontaneous, reversible dissolution and re-nucleation in response to small fluctuations in the driving forces.

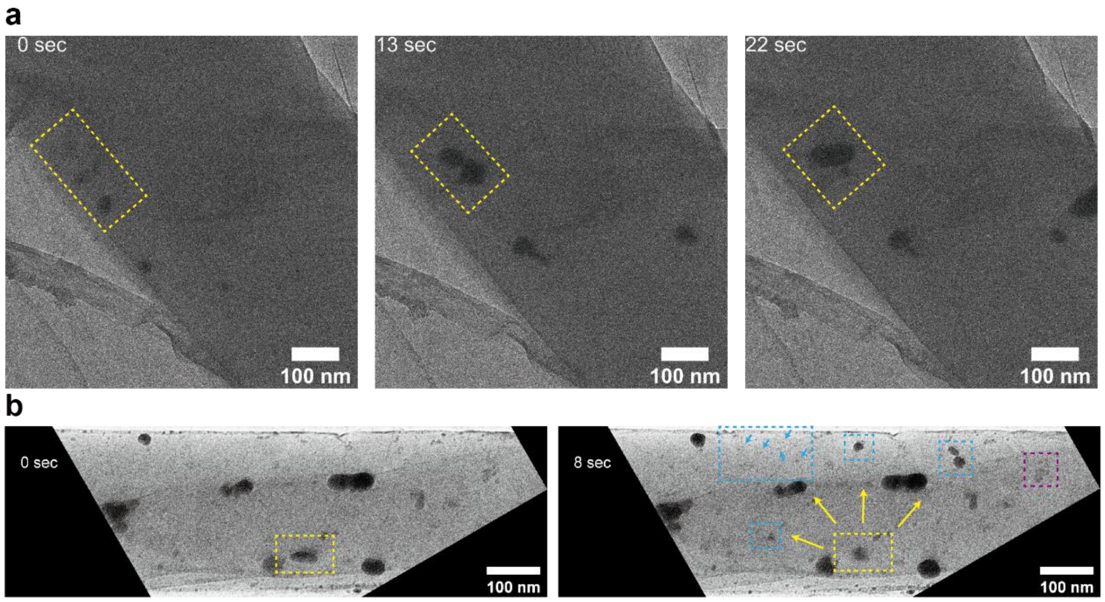

Figure S5. Disassembly, reassembly, fusion, and coexistence of oligomers and the dense phase. (a) Clusters and a dense phase (yellow dashed box at 0 s) first fuse/grow into two dense phases (yellow dashed boxes at 13 s), which subsequently fuse into a larger dense phase (yellow dashed box at 22 s). Corresponding to **Movie S8**. **(b)** A dense phase (outlined by a yellow dashed box at 0 s) disassembles and reassembles, yielding several smaller dense phases (blue dashed boxes at 8 s) and oligomers (blue arrows within the blue dashed boxes at 8 s), with clusters (purple dashed box) coexisting. Corresponding to **Movie S9**. Scale bar: 100 nm.

11. Discussion on multi-step density transition and NNT

According to CNT, biomolecule clusters of radius R form due to thermal fluctuations with free energy given by $\Delta G(r) = 4\pi r^2\gamma + \frac{4}{3}\pi r^3\epsilon$, depending on surface tension γ and free energy per unit volume ϵ. In order to calculate critical radius r^*, differentiate $\Delta G(R)$with respect to r and set the derivative to zero:

$$\frac{\mathrm{d}\Delta G(r)}{\mathrm{dr}} = 8\pi r\gamma - 4\pi r^2\epsilon = 0$$

which gives $r^* = \frac{2\gamma}{\epsilon}$. Under the ideal approximation, $\epsilon \approx \rho\mathrm{kTln}S$, where S is the supersaturation and ρ is the dense-phase number density. In our experiments we take 50 mM, since our estimate for the dense-phase concentration is 19–110 mM. Taking $T = 300$ K and $\ln S = 0.01$, we can estimate ϵ:

$$\epsilon \approx \rho k\mathrm{Tln}S \approx 50\,m^{-3} \times 6.02 \times 10^{23} \times 4.11 \times 10^{-21} J \times 0.01 \approx 1200\,J/m^3$$

For LLPS droplets, studies suggest the interfacial tension is about $5 \times 10^{-6}\,N/m$ [55], substituting into $r^* = \frac{2\gamma}{\epsilon}$, gives $r^* = \frac{2\times5\times10^{-6}}{1200} \approx 8.3\,nm$.

Different from CNT, our experiments reveal a multistep pathway and threshold behavior: small oligomers and low-density network-like clusters form and can persist even under subsaturated conditions, and only when a cluster's occupancy exceeds a threshold of about 20 molecules do we observe a rapid densification into a compact, near-spherical high-density phase (with internal concentrations on the order of 19–110 mM). More importantly, the initial size of dense phase is 8.6 nm, 5.5 nm, 5.6 nm, 4.8 nm, 5.8 nm, 10.0 nm, 8.0 nm, 5.9 nm, 10.0 nm. Thus, the decisive event is not set by size alone but by a coupling between size and interaction/conformational rearrangements; the early clusters exist in sub-saturated solution are neither CNT-like. In this way we believe our experiments present a two-step NNT process.

Furthermore, the LLPS phenomenon is commonly described using the classical mean-field Flory–Huggins theory. [56]:

$$\frac{F}{k_B T} = \frac{\Phi}{N}\ln\Phi + (1-\Phi)\ln(1-\Phi) + F_{int}$$

In this equation, F stands for the free energy, Φ is the volume fraction, N is the chain length and F_{int} represents the interaction free energy.

When interactions are strong—reflected by a very negative effective macromolecule–solvent interaction parameter χ, a large electrostatic coupling constant α, or a high charge density σ—the system is expected to undergo a discontinuous demixing into two coexisting liquid phases, first order phase transition. The breadth of this biphasic region is governed by the relative strengths of these interactions, which typically depend on factors such as temperature, pH, the chemical functionality of the macromolecules, and the salt (ionic strength) in solution. In our manuscript, we observed a nonclassical nucleation process in protein solution at the first stage of LLPS. Although it departs from CNT, NNT is merely an alternative kinetic pathway to the same first-order phase transition as CNT; both are first order phase transition. In contrast, second-order phase transitions proceed without nucleation [57]. Therefore, the density transition should be classified as a first-order phase transition.

SI References

1. Esteban-Hofer L, Emmanouilidis L, Yulikov M *et al*. Ensemble structure of the N-terminal domain (1–267) of FUS in a biomolecular condensate. *Biophys J* 2024; **123**: 538-54.
2. Bates GP, Kang J, Lim L *et al*. A unified mechanism for LLPS of ALS/FTLD-causing FUS as well as its modulation by ATP and oligonucleic acids. *PLoS Biol* 2019; **17**: 1-33.
3. Boyko S, Surewicz K, andSurewicz WK. Regulatory mechanisms of tau protein fibrillation under the conditions of liquid–liquid phase separation. *Proc Natl Acad Sci USA* 2020; **117**: 31882-90.
4. Nagamanasa KH, Wang H, andGranick S. Liquid-cell electron microscopy of adsorbed polymers. *Adv Mater* 2017; **29**: 1703555.
5. Wang H, Xu Z, Mao S *et al*. Experimental guidelines to image transient single-molecule events using graphene liquid cell electron microscopy. *ACS Nano* 2022; **16**: 18526–37.
6. Cheng B, Ye E, Sun H *et al*. Deep learning-assisted analysis of single molecule dynamics from liquid-phase electron microscopy. *Chem Commun* 2023; **59**: 1701-4.
7. Egerton RF, Li P, andMalac M. Radiation damage in the TEM and SEM. *Micron* 2004; **35**: 399-409.
8. Langmore JP and Smith MF. Quantitative energy-filtered electron microscopy of biological molecules in ice. *Ultramicroscopy* 1992; **46**: 349-373.
9. Fritsch B, Malgaretti P, Harting J *et al*. Precision of radiation chemistry networks: Playing jenga with kinetic models for Liquid-Phase Electron Microscopy. *Precis Chem* 2023; **1**: 592-601.
10. Schneider NM, Norton MM, Mendel BJ *et al*. Electron–water interactions and implications for Liquid Cell Electron Microscopy. *J Phys Chem C* 2014; **118**: 22373-82.
11. Woehl TJ, Moser T, Evans JE *et al*. Electron-beam-driven chemical processes during liquid phase transmission electron microscopy. *MRS Bulletin* 2020; **45**: 746-753.
12. Egerton RF. Mechanisms of radiation damage in beam-sensitive specimens, for TEM accelerating voltages between 10 and 300 kV. *Microsc Res Tech* 2012; **75**: 1550-1556.
13. Smith JW, Carnevale LN, Das A *et al*. Electron videography of a lipid–protein tango. *Sci Adv* 2024; **10**: eadk0217.
14. Wu H, Friedrich H, Patterson JP *et al*. Liquid-Phase Electron Microscopy for soft matter science and biology. *Adv Mater* 2020; **32**: 2001582.
15. Smith JW and Chen Q. Liquid-phase electron microscopy imaging of cellular and biomolecular systems. *J Mater Chem B* 2020; **8**: 8490-506.
16. Pu S, Gong C, andRobertson AW. Liquid cell transmission electron microscopy and its applications. *R Soc Open Sci* 2020; **7**: 191204.
17. Liao H-G and Zheng H. Liquid cell transmission electron microscopy. *Annu Rev Phys Chem* 2016; **67**: 719-47.

18. Mirsaidov U, Patterson JP, andZheng H. Liquid phase transmission electron microscopy for imaging of nanoscale processes in solution. *MRS Bulletin* 2020; **45**: 704-712.
19. Parent LR, Bakalis E, Proetto M *et al*. Tackling the challenges of dynamic experiments using liquid-cell transmission electron microscopy. *Acc Chem Res* 2017; **51**: 3-11.
20. Ou Z, Wang Z, Luo B *et al*. Kinetic pathways of crystallization at the nanoscale. *Nat Mater* 2019; **19**: 450-5.
21. Yuk JM, Park J, Ercius P *et al*. High-Resolution EM of colloidal nanocrystal growth using graphene liquid cells. *Science* 2012; **336**: 61-64.
22. Li JY, Zhang DY, Mao S *et al*. Single molecule imaging with Liquid Phase Electron Microscopy. *Chin J Chem* 2023; **41**: 679-84.
23. Li J-Y, Sun H, andWang H. Imaging biomacromolecules in action with Liquid-Phase Electron Microscopy. *Trends Chem* 2024; **6**: 281-4.
24. Glaeser RM, Sonani RR, andEgelman EH. Liquid phase biological electron microscopy: Many published results and claimed benefits are fantasy, not fact. *Microsc Microanal* 2025; **31**: 1-7.
25. Fugallo G, Cepellotti A, Paulatto L *et al*. Thermal conductivity of graphene and graphite: Collective excitations and mean free paths. *Nano Lett* 2014; **14**: 6109-14.
26. Cao M, Xiong DB, Yang L *et al*. Ultrahigh electrical conductivity of graphene embedded in metals. *Adv Funct Mater* 2019; **29**: 1806792.
27. Cho H, Jones MR, Nguyen SC *et al*. The use of graphene and its derivatives for liquid-phase transmission electron microscopy of radiation-sensitive specimens. *Nano Lett* 2016; **17**: 414-20.
28. Meyer JC, Eder F, Kurasch S *et al*. Accurate measurement of electron beam induced displacement cross sections for single-layer graphene. *Phys Rev Lett* 2012; **108**: 196102.
29. Wang H, Li B, Kim Y-J *et al*. Intermediate states of molecular self-assembly from liquid-cell electron microscopy. *Proc Natl Acad Sci USA* 2020; **117**: 1283-92.
30. Keskin S and de Jonge N. Reduced radiation damage in transmission electron microscopy of proteins in graphene liquid cells. *Nano Lett* 2018; **18**: 7435-40.
31. Touve MA, Carlini AS, andGianneschi NC. Self-assembling peptides imaged by correlated liquid cell transmission electron microscopy and MALDI-imaging mass spectrometry. *Nat Commun* 2019; **10**: 4837.
32. Park J, Jeong H, Noh N *et al*. Single-molecule graphene Liquid Cell Electron Microscopy for instability of intermediate amyloid fibrils. *Adv Mater* 2023; **36**: 1-8.
33. Wang H, Nagamanasa KH, Kim Y-J *et al*. Longer-lasting electron-based microscopy of single molecules in aqueous medium. *ACS Nano* 2018; **12**: 8572-8578.
34. Filice M, Guisan JM, Terreni M *et al*. Regioselective monodeprotection of peracetylated carbohydrates. *Nat Protoc* 2012; **7**: 1783-96.

35. Li J-Y, Liu F, Xu J *et al*. The ergodicity question when imaging DNA conformation using liquid cell electron microscopy. *Proc Natl Acad Sci USA* 2024; **121**: e2314797121.
36. Li J-y, Wang Z-b, Xu Z-p *et al*. Modes of nanodroplet formation and growth on an ultrathin water film. *J Phys Chem B* 2024; **128**: 3732-41.
37. Park J, Koo K, Noh N *et al*. Graphene liquid cell electron microscopy: progress, applications, and perspectives. *ACS Nano* 2021; **15**: 288-308.
38. Verch A, Pfaff M, andde Jonge N. Exceptionally slow movement of gold nanoparticles at a solid/liquid interface investigated by scanning transmission electron microscopy. *Langmuir* 2015; **31**: 6956-6964.
39. Kang S, Kim J-H, Lee M *et al*. Real-space imaging of nanoparticle transport and interaction dynamics by graphene liquid cell TEM. *Sci Adv* 2021; **7**: 1-10.
40. Lu J, Aabdin Z, Loh ND *et al*. Nanoparticle dynamics in a nanodroplet. *Nano Lett* 2014; **14**: 2111-5.
41. Villegas JA, Heidenreich M, andLevy ED. Molecular and environmental determinants of biomolecular condensate formation. *Nat Chem Biol* 2022; **18**: 1319-29.
42. Krainer G, Welsh TJ, Joseph JA *et al*. Reentrant liquid condensate phase of proteins is stabilized by hydrophobic and non-ionic interactions. *Nat Commun* 2021; **12**: 1085.
43. Qamar S, Wang G, Randle SJ *et al*. FUS phase separation is modulated by a molecular chaperone and methylation of arginine cation-π interactions. *Cell* 2018; **173**: 720-734.e15.
44. Murthy AC, Dignon GL, Kan Y *et al*. Molecular interactions underlying liquid−liquid phase separation of the FUS low-complexity domain. *Nat Struct Mol Biol* 2019; **26**: 637-48.
45. Jumper J, Evans R, Pritzel A *et al*. Highly accurate protein structure prediction with AlphaFold. *Nature* 2021; **596**: 583-589.
46. Anandakrishnan R, Aguilar B, andOnufriev AV. H++ 3.0: automating pK prediction and the preparation of biomolecular structures for atomistic molecular modeling and simulations. *Nucleic Acids Res* 2012; **40**: W537-41.
47. Götz AW, Williamson MJ, Xu D *et al*. Routine microsecond molecular dynamics simulations with amber on gpus. 1. Generalized born. *J Chem Theory Comput* 2012; **8**: 1542-55.
48. Tian C, Kasavajhala K, Belfon KAA *et al*. ff19sb: Amino-acid-specific protein backbone parameters trained against quantum mechanics energy surfaces in solution. *J Chem Theory Comput* 2019; **16**: 528-52.
49. Izadi S, Anandakrishnan R, andOnufriev AV. Building water models: A different approach. *J Phys Chem B* 2014; **5**: 3863-71.
50. Zhou H, Wu G, Zhang Z *et al*. Data-driven design of random heteropolypeptides as synthetic polyclonal antibodies. *J Am Chem Soc* 2025; **147**: 21077-88.
51. Isihara A. Probable distribution of segments of a polymer around the center of gravity. *J Phys Soc Jpn* 1950; **5**: 201a.
52. Shaheen ME, Ghazy AR, Kenawy E-R *et al*. Application of laser light scattering

to the determination of molecular weight, second virial coefficient, and radius of gyration of chitosan. *Polymer* 2018; **158**: 18-24.
53. George A and Wilson WW. Predicting protein crystallization from a dilute solution property. *Acta Crystallogr, Sec. D: Biol Crystallogr* 1994; **50**: 361-5.
54. Wolde PRt and Frenkel D. Enhancement of Protein Crystal Nucleation by Critical Density Fluctuations. *Science* 1997; **277**: 1975-1978.
55. Jawerth L, Fischer-Friedrich E, Saha S *et al*. Protein condensates as aging Maxwell fluids. *Science* 2020; **370**: 1317-1323.
56. Xu Z, Wang W, Cao Y *et al*. Liquid-liquid phase separation: Fundamental physical principles, biological implications, and applications in supramolecular materials engineering. *Supramol Mater* 2023; **2**: 100049.
57. Binder K. Theory of first-order phase transitions. *Rep Prog Phys* 1987; **50**: 783-859.
